# Scaffolds of Macroporous Tannin Spray With Human-Induced Pluripotent Stem Cells

**DOI:** 10.3389/fbioe.2020.00951

**Published:** 2020-10-15

**Authors:** Yongbo Yang, Soliman Abdalla

**Affiliations:** ^1^Department of Orthopedics (Spine), Xinxiang Central Hospital, Xinxiang City, China; ^2^Department of Physics, Faculty of Science, King Abdulaziz University, Jeddah, Saudi Arabia

**Keywords:** solid scaffolds, vivo-stem cells, macroporous tannin spray, bone engineering, tannin composite

## Abstract

Skeletal defects resulting from trauma and disease represent a major clinical problem worldwide exacerbated further by global population growth and an increasing number of elderly people. As treatment options are limited, bone tissue engineering opens the doors to start an infinite amount of tissue/bone biomaterials having excellent therapeutic potential for the management of clinical cases characterized by severe bone loss. Bone engineering relies on the use of compliant biomaterial scaffolds, osteocompetent cells, and biologically active agents. In fact, we are interested to use a new natural material, tannin. Among other materials, porous tannin spray-dried powder (PTSDP) has been approved for human use. We use PTSDP as reconstructive materials with low cost, biocompatibility, and potential ability to be replaced by bone *in vivo*. In this study, macro PTSDP scaffolds with defined geometry, porosity, and mechanical properties are manufactured using a combination of casting technology and porogen leaching, by mixing PTSDP and hydroxyapatite Ca10(PO4)6(OH)2 with polyethylene glycol macroparticles. Our results show that the scaffolds developed in this work support attachment, long-term viability, and osteogenic differentiation of human-induced pluripotent stem cell-derived mesenchymal progenitors. The combination of select macroporous PTSDP scaffolds with patient-specific osteocompetent cells offers new opportunities to grow autologous bone grafts with enhanced clinical potential for complex skeletal reconstructions.

## Introduction

We have recently reported on new natural organic materials: Derivatives of condensed poly flavonoid tannins are characterized by ease in manufacturing and can be used in different applications (Celzard et al., 2013, 2015; Lacoste et al., 2013; Basso et al., 2014b; Lagel et al., 2016; Al-Marzouki et al., 2017; Abdalla et al., 2018; Guo et al., 2018). However, the medical applications of these materials are still poor. In addition, we have noticed that the mechanical properties of these derivatives can vary within a vast range of properties, starting from very soft materials such as in reference (Guo et al., 2018) up to very hard materials such as automobile brake pads (Lagel et al., 2016; Al-Marzouki et al., 2017) depending on their method of preparation. In addition, these materials can be prepared with different porosity ratios. In fact, scientists are interested to treat bone faults due to the continuous increase of aged persons within the continuous global public growth who have expanded life probability to be around two billion by 2050 (Clegg et al., 2013). In general, the continuous need for bone-tissue (BT) substitutive techniques rises continuously by time. However, to treat BT defects, scientists use techniques essentially transplantation of BT-implantations. These BT-implantations can improve the essential characteristics of tissue; however, they do not succeed to give suitable solutions to different medical problem.

As opposite, biomaterials can be used in bone-substitutes by culturing osteocompetent cells onto flexble-biomaterials, offering the possibility to grow unlimited amounts of tissue products with enhanced regenerative potential and broader clinical use (Oreffo and Triffitt, 1999).

In fact, with porous tannin spray-dried powder (PTSDP), we are interested to study and characterize some natural materials to give new different applications (Tondi and Pizzi, 2009; Li et al., 2012; Amaral-Labat et al., 2013; Pizzi et al., 2013; Basso et al., 2014a, 2015; Abdalla et al., 2015; Konai et al., 2015). The PTSDP tannin are prepared in such a way to resemble ceramic as biomaterials for bone engineering applications, here, and the tannin has been prepared as PTSDP. These materials are FDA-approved for human application (U.S. Food and Drug Administration, 2019). PTSDP is a natural material with a chemistry that fits the mineral phase of bones, can be fabricated into different shape at a low cost, and is bio compatible and osteotransductive, i.e., it can be integrated and reintegrated by bone tissue after indoctrination in bone-patients. Interfacing osteocomponent stem cells onto PTSDP scaffolds holds the potential to enhance the healing properties of these materials, and no studies over the last years have reported such attempts. However, currently available PTSDP scaffolds lack macroporosity, a critical feature that allows cell infiltration, communication, and growth *in vitro*-*vivo*. Scaffold porosity can also facilitate bone in growth and remodeling in therefore maximizing the therapeutic potential of tissue-engineered products. Specifically in bone engineering, porosity is crucial to develop biomaterial scaffolds, mimicking the architecture of the native bone tissue.

In the present study, macro-PTSDP scaffolds are manufactured as we have described elsewhere (Abdalla et al., 2018). And scaffold macroporosity are fabricated by variation and controlling both the diameter and number of polyethylene glycol particles (PEGPs), in such a way that one can get the most suitable macroporosity for scaffold; then, one can characterized them for other properties such as: chemical, structural, degree of porosity, their mechanical features, …etc. This is seeded with human-induced pluripotent stem cell (hiPSC)-derived mesenchymal progenitors (scamps) to assess their potential for bone engineering applications. hiPSCs can be derived using minimally invasive procedures from small tissue samples (Takahashi et al., 2007; Yu et al., 2009; Kim, 2015), displaying broad differentiation potential toward all cells constituting the bone tissue. They proliferate extensively and open the possibility to grow a large amount of functional tissue substitutes for tautologous applications.

A common trend in tissue engineering is to use bio-mimetic substrates derived from decellularized tissues, because they display cues necessary to guide functional tissue regeneration. The present data show that the macro-PTSDP scaffolds developed in this study support attachment, long-term viability, and pathogenic differentiation of scamps. This performs to a similar extent as bio-mimetic decellularized bone scaffolds (DBSs) and highlights the potential of these scaffolding materials in the engineering of functional bone grafts for personalized applications. It is worth noting that a number of sections in the present work can be accessed on our previous patent (Abdalla et al., 2018), which is found in the link: https://patents.justia.com/patent/10155069.

## Results and Discussion

### Scaffold Contour-Field and Its Chemical Characteristics

Water molecules, which are found between crystals-edges, when one form the PTSDP-cement, can affect the formation of scaffolds, and in particular, they lead to micro-porosity with tinn diameter not more than 100 μm.

**FIGURE 1:**
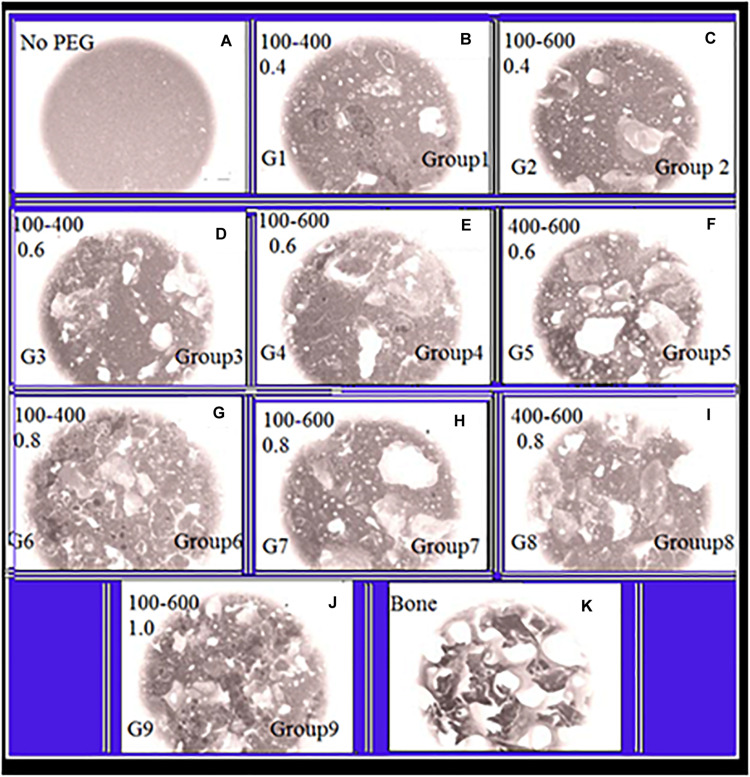
Panels **(A–K)** are SEM-illustrations of macroporous scaffold with different amounts of PEGPs as indicated below: **(A)** Reference sample with zero PEGPs, Group1 (G1), **(B)** 0.4 weight-ratio (WR) of PEGPs with 100–400 μm diameter PEGPs. **(C)** (G-2): 0.4 WR of PEGPs with 100–600 μm diameter PEGPs. **(D)** (G-3): 0.6 WR of PEGPs with 100–400 μm diameter PEGPs. **(E)** (G-4): 0.6 WR of PEGPs with 100–600 μm diameter PEGPs. **(F)** (G-5): 0.6 WR of PEGPs with 400–600 μm diameter PEGPs. **(G)** (G-6): 0.8 WR of PEGPs with 100–400 μm diameter PEGPs. **(H)** (G-7): 0.8 WR of PEGPs with 100–600 μm diameter PEGPs. **(I)** C – (G-8): 0.8 WR of PEGPs with 400–600 μm diameter PEGPs. **(J)** (G-9): 1.0 WR of PEGPs with 100–600 μm diameter PEGPs. **(K)** Real decellularized/bone-scaffold.

Figure 1A shows that the control group (without PEGPs) has fine nameless topography with only tinny cavities circulating along the scaffold exterior. The amount of unreacted PTSDP appears to increase with increasing the PEG content from 0.4 to 1 g of PEG/g of cement. This occurs for each PEGP size, and the amount of the unreacted PTSDP decreases with increasing the size of PEGPs for any PEG content, except for the scaffold groups manufactured using 0.8 g of PEG/g of cement (groups 6–8 Figures 1G–I). At the lowest amount of PEG content (0.4 g/g), the 100–600 μm groups contain significantly less unreacted PTSDP than 100–400 or 400–600 μm groups (*P* < 0.001), while at larger PEG amounts (0.8 g/g) the 400–600 μm scaffolds contain less unreacted PTSDP than 100–600 μm (*P* = 0.05^∗^) or 100–400 μm (*P* = 0.008^∗^) groups. Between groups with different particle size, the increase in unreacted PTSDP is particularly pronounced in the samples manufactured using the 100–600 μm PEGPs (group 2, 4, and 7). This results in a 382% (*P* < 0.001) increase in unreacted PTSDP as the amount of PEG increases from 0.4 g to 0.8 g/g, compared to a 13 and 20% increase for the samples manufactured using 100–400 and 400–600 μm PEGPs, respectively. PEG is hydrophilic, readily absorbs water, and typically creates a hydration shell containing 1–4 molecules of water per molecule of PEG (Crupi et al., 1999). When the size of the PEGP decreases, the total surface area will commensurately increase for each content category. Water should be gotten out of the PTSDP to form a hydration shell on the surface of PEGPs, with smaller particles pulling more water from the reaction than larger ones. For any given PEG content, the use of larger PEGP size (400–600 μm compared to 100–600 μm) reduced the total porosity, which is consistent with the theory that smaller particles pull more water from the reaction, thus increasing the scaffold porosity (Chen et al., 2018; Lee et al., 2018; Zhang et al., 2018). During the PTSDP dissolution/precipitation reaction, PEG can also directly interfere with the precipitation of dissolved PTSDP onto PEGPs. XRD analysis used in order to verify the leaching of PEGPs from cement scaffolds. Thus, PEG interactions of PEG and PTSDP are poorly characterized (Natarajan et al., 2012; Vu et al., 2015; Lin et al., 2019). Figures 1A–L show that PEG can reduce the particle size of PTSDP 100-fold (584 to 6 μm in diameter) and affect the properties of PTSDP particles when as little as 0.4% is adsorbed onto the surface which is directly adsorb onto the surface of PTSDP particles.

We have checked the leaching of PEGPs from cement scaffolds by XRD experimental investigation. This directly influences the surface area of PTSDP available for precipitation to occur. Thus, PEG can directly adsorb onto the surface of PTSDP particles, thereby influencing the surface area of PTSDP available for precipitation to occur. The spectrogram shows no peaks typical of pure PEG in all cement scaffold groups. The complete removal of PEGPs indirectly indicates the presence of a network of interconnected pores forming inside the materials, which is critical to promote optimal tissue regeneration *in vitro* and *in vivo*.

### Mechanical Characteristics and Porosity as a Function of PEGPs Content

In the present study, we use micro-computed tomography (μCT) in order to investigate the scaffold porosity and its pycnometry.

**FIGURE 2:**
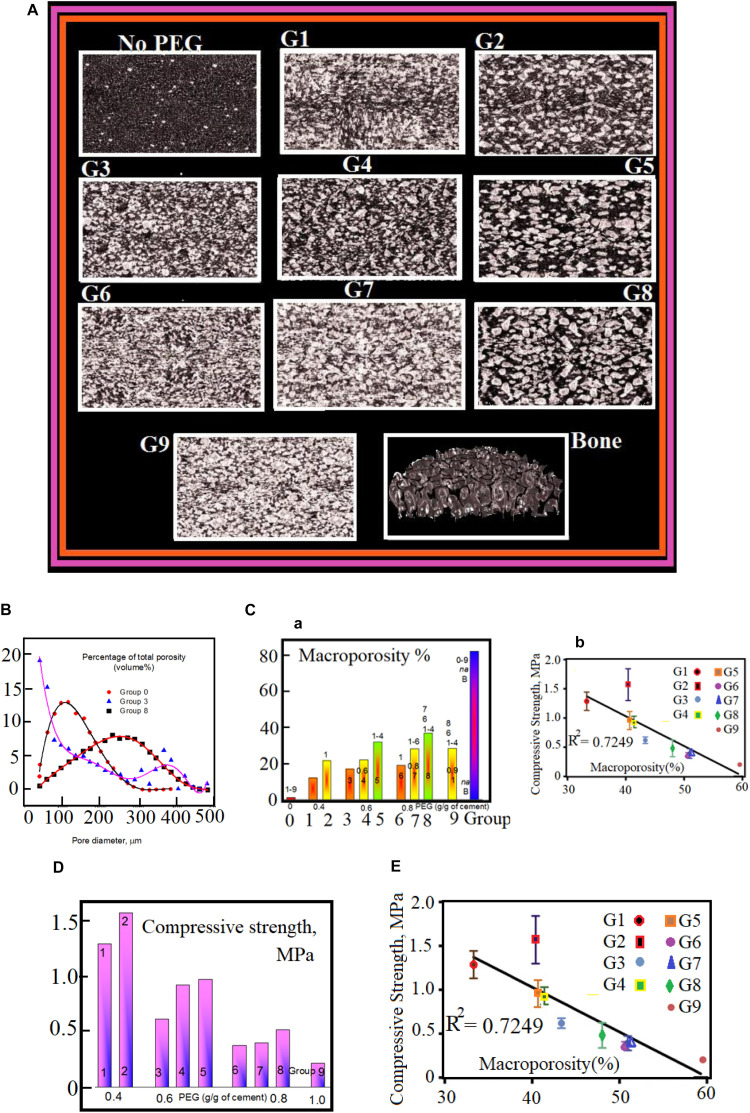
**(A)** μCT images of DB tissue and cement scaffolds. The corresponding content of PEGPs and the diameter of PEGPs in micrometer with 1 mm as scale bar are as following: (No PEG): zero – (G1): 0.4 and 100–400 (G2) 0.4 and 100–600 – (G3) 0.6, and 100–400 – (G4) 0.6 and 100–600 – (G5) 0.6 and 400–600 – (G6) 0.8 and 100–400 – (G7) 0.8 and 100–600 – (G8) 0.8 and 400–600 (I) (G9) 1.0 100–400; Bone. **(B)** Pore distribution of cement scaffolds and DB as measured by μCT. **(C–a)** Macroporosity rate (percent) for each cement scaffolds. The experimental data are measured by μCT. Panel **(C–b)** shows the decrease of compressive strength, for the nine scaffold groups, as a function of macroporosity with *R*^2^ = 0.7249. **(D)** Compulsive power (Compressive-strength) as a function of PEGPs. The compulsive power varies inversely with the content of PEGPs: The compulsive power rises when total quantity of PEGPs decreases. The mean average of the experimental data for the groups 5 and 6 with standard deviation ± *P* < 0.05. Panel **(E)** shows the decrease of compressive strength, for the nine scaffold groups, as a function of macroporosity with *R*^2^ = 0.7249.

All groups with PEGPs have relatively lager pores (with diameter >100 μm). This is obtained from the close investigation of data shown in Figure 2A. These data are in direct opposition against the control group (having no PEGPs). Because the content of PEGPs and their diameter affect the level of interdependence, i.e., this level rises with the content and diameter of PEGPs. One can see the pore diameter distribution in Figure 2B for the samples. All groups show various portraits of porosity. For example, the pore diameter distribution lies in the range from 40 μm to 300 μm when 100–400 μm PEGPs are used with a curve having a maximum in the range 100 μm < pore diameters < 120 μm. While this pore diameter distribution lies in the range from 40 μm to 480 μm when 100–600 μm PEGPs are used with a curve having a maximum in the range 100 μm < pore diameters < 160 μm. The last curve shows that the pore diameter distribution lies in the range from 40 μm to 480 μm when one uses 400–600 μm PEGPs with a curve having a maximum in the range 220 μm < pore diameters < 320 μm.

The percentage of macroporosity appears highest in the scaffolds manufactured with PEG content of 0.8 g/g and particle size 400–600 μm, which is significantly higher than all groups except groups 5 and 9. The percentage of macroporosity is around 80% for DBSs.

The chemical composition and physical parameters (porosity, pore size, etc.) greatly influence the mechanical properties of biomaterial scaffolds. For a given chemical composition, an increase in scaffold porosity results in decreased mechanical stiffness (Jeonga and Hollister, 2010; Chen et al., 2014). In this study, both the particle size and content of PEG affected the porosity, as well as the pore distribution of the scaffolds, resulting in PTSDP scaffold groups characterized by variable compressive strength (CS), which range from 0.20 ± 0.03 to 1.57 ± 0.27 MPa for samples manufactured using PEGPs as shown in Figure 2D. The reduction in CS associated with increasing porosity has been observed in other ceramics (Xu et al., 2015; Khaledi et al., 2018, 2019). Overall, the CS appears to decrease as the amount of PEG increases regardless of the PEGP size.

Cellular attachment within the scaffold is highly affected by the pore diameter, its porosity, interconnections, and distribution. In average terms, the pore size lies in the range between 100 to 350 μm. Both mechanical and biological properties of the scaffold are affected with the pore size. For example, when pores are too small, cell migration will face difficulties with good stability of the scaffold. However, when pores are too large, cell migration will be free but with poor stability of the scaffold.

Homogeneous distribution affects the porosity and interconnections through the matrix.

One can built a good scaffold when taking the optimum conditions between mechanical stability (need small pores) and biological properties (need large pores).

Groups with a narrower particle size distribution and larger particle size are consistently stronger. The samples manufactured with diameters in the range 400–600 μm PEGPs are stronger than the samples having 100–400 or 100–600 μm. Similarly, the samples having 100–600 μm are stronger than those having 100–400 μm.

The larger pore diameter is expected to produce larger mean inter-pore strut thickness. Al-Munajjed et al. (2008) have observed this behavior in some hyaluronan-collagen scaffolds and this can explain the difference in mechanical strength observed (Stern et al., 2007; Al-Munajjed et al., 2008; Pennella et al., 2013; Park et al., 2015). Impressive fact is that all manufactured samples with no PEGPs has a compulsive power nearly doubled of that DB: For the fabricated cement-scaffolds, the CS is about 15.2 ± 2 MPa, while the CS of the bone-scaffolds is about 6.24 ± 2.02 MPa. Here, the dependence of the porosity on mechanical features are well manifested. This dependence supports the fact that CS of scaffolds with PEGPs have poor values compared with the samples having no PEGPs (control group). A curve plotted between the CS against the macro- and total (= macro- + intrinsic-) porosity reveals that the R 0.7358 and 0.504, respectively. This indicates a strong relationship between the macroporosity and the ultimate CS of the scaffolds.

In general, the macroporosity of all scaffolds (Figure 2C) varies in the range from 10 to 40%. The CS of the cement scaffolds is highly affected with the variations of particles size distribution. In general, the CS seems to attenuate with the PEGPs content whatever the size of PEGPs (Figure 2C). We have noticed that groups with larger diameter are more stable and strongly built. Cement scaffolds with no PEGPs have CS equals 15.18 ± 2.7 MPa; they are twice stronger than those with as DBSs (6.3 ± 1.14 MPa). The reason for that is that the mechanical properties are highly affected by the scaffold macroporosity. Figure 2E shows the net decrease of CS as a function of scaffold porosity. This decrease indicates the strong correlation between the macroporosity and the CS of the investigated scaffolds.

Future systematic studies are necessary to fully understand the relation between structural parameters and mechanical compliance so that optimal scaffolds can be developed for specific biomedical applications.

### Derivation and Characterization of iPSC-MPs

**FIGURE 3:**
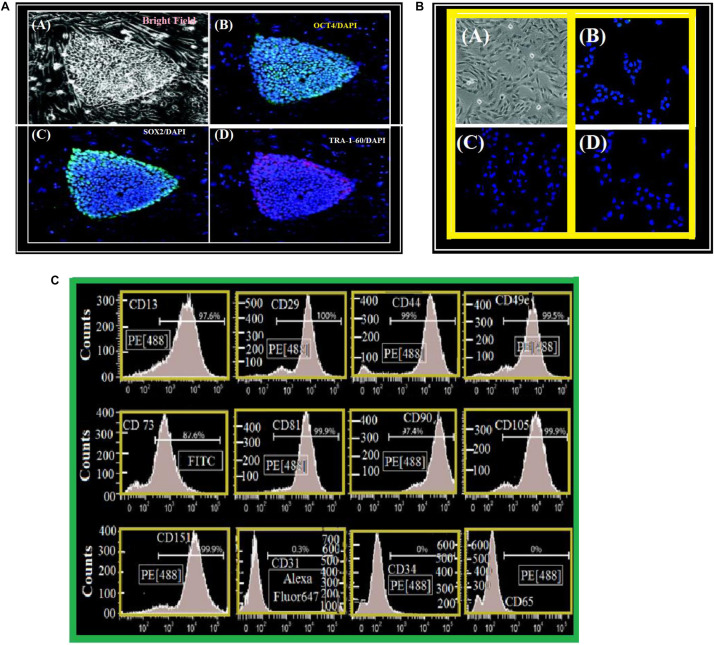
**(A)** Cell line derivation and characterization. (A) Undifferentiated 1013A human-induced pluripotent stem cell line (bright field). (B–D) Human-induced pluripotent stem cell line is positive – OCT4 (B), SOX2 (C), and TRA-1-60 (D). We stained nuclei with DAPI/Blue. (A) Scale bar = 50 μm. (B–D) Scale bar = 200 μm. **(B)** Cell line characterization. (A) fibroblastic-mesenchymal cells as seen in a bright field microscope. (B–D) Syllable structure of 1013A-derived/mesenchymal “1013A-MP-progenitors” at transit 4 (P4). Negative-cells for OCT4 (B), SOX2 (C) and TRA-1-60 (D). We have used DAPI to stain the nuclei. Scale bar = 50 μm. **(C)** A surface antigen-profile of primary mesenchymal-cells obtained by Flow-cytometry measurements of 1013A-MP.

Mesenchymal progenitors were derived from the pluripotent stem cell line 1013A as previously described (Abdalla et al., 2018). Colonies of 1013A cells display typical iPSC morphology and are positive for the pluripotency markers OCT4, SOX2, and TRA-1-60 as shown in Figure 3A. In contrast, derived mesenchymal progenitors are negatively charged for TRA-1-60+, SOX2, and OCT4. They act with an internal construction having fibroblastic appearance with (CD 31-CD 34-CD 45) markers. However, they show strong style of markers Figure 3C.

#### Flow-Cytometry and Seeding of iPSC-MPs

**FIGURE 4:**
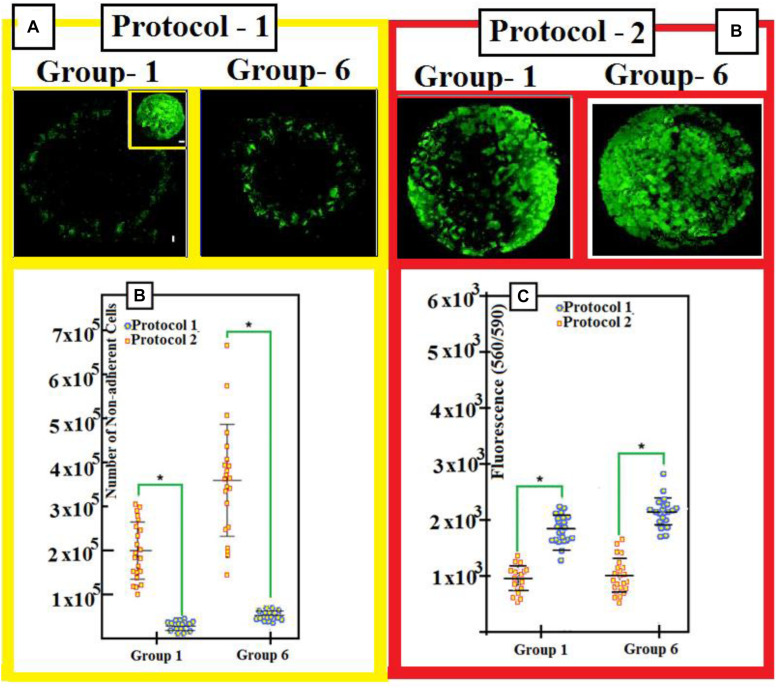
**(A)** Three days after seeding, toper view is a patchwork-illustrating cell seeding (with protocols 1 and 2) into the scaffolds. Upper illustration shows mosaic-arts with the scaffold cellularity 3 days after seeding. We use both protocols 1 and 2. We stain cells with Green-calcian. Scale bar = 1 mm. We put in the inset: Cell density and distributions on DBSs. Scale bar = 1 mm. **(B)** We use protocols 1 and 2 for groups 1 and 6: After we seed for 1 day, the number of non-adherent cells is shown after seeding for 1 day. **(C)** Calibration of number of adherent-cells 1-day after we seed with protocol 1 and protocol 2. In order to compare data, we use a two-tailed un-paired *t*-test. Experimental results with mean averages ± standard deviation (n = 22, *P* < 0.05). We put an asterisk in order to represent important changes between protocol 1 and protocol 2.

We have remarked that the cells have fibers shapes (rods in Figures 9a–d). They are negatively charged for pluri-potency [-OCT4-SOX2-TRA, 1, 60] and hematopoietic [-CD31-CD34-CD45] markers. However, they show strong expression of markers (Figure 3C) [-CD13-CD29-CD44-CD49e-CD73-CD81-CD90-CD105-and-CD151]. Figure 4 shows two protocols used to increase the number of attached cells (NACs). In protocol 2, the NACs is higher than in protocol 1; and they scatter around the surface of the scaffolds. Figure 4B shows a net increase of cell attachment when reducing number of number of non-adherent cells. This figure shows that there is an increase in fluorescence measured, followed to the incubation with PrestoBlue^®^ reagent. However, the cells-seeding efficiency is the same for both protocols, independent on the used-group.

#### Cell Viability

**FIGURE 5:**
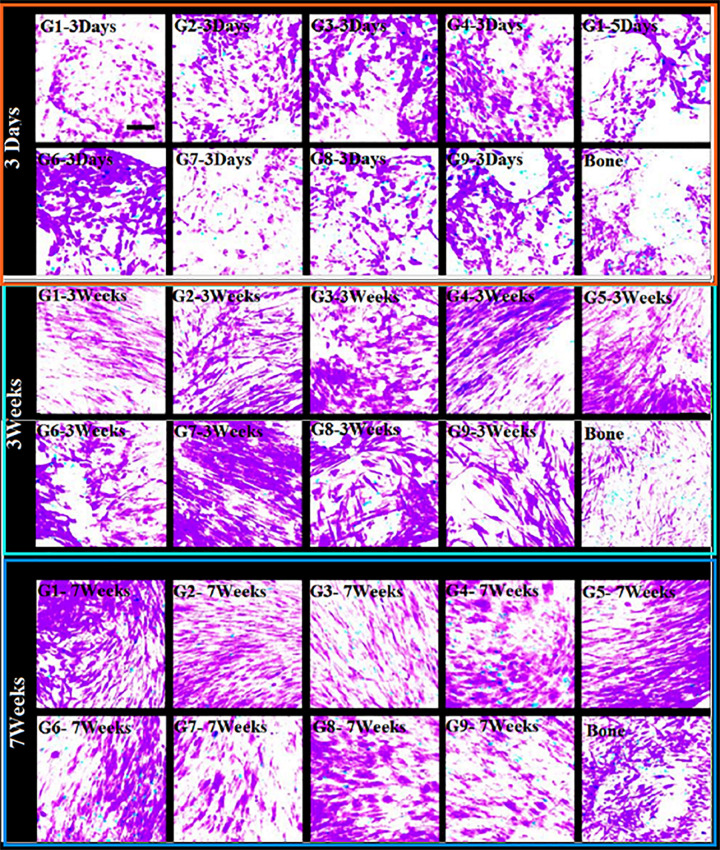
Cell-growth (cell activity). Life-durability (after seeding) of 1013A-derived mesenchymal progenitors, 1013A-MPs. This is measured under osteogenic situations on cement-scaffolds and decellularized-bone: 3 days, 3 weeks, and 7 weeks. We denote by red color dead-cells while green color stands for live-cells staining of cell/scaffold builds shows identical cell-activity between scaffold-groups and decellularized-bone. Scale bar = 100 μm.

We have estimated cell viabilities through dead/live-assays. Figure 5 shows that independent on the scaffold group, cells behave with similar viability when they are cultured under osteogenic states for periods: 3 days, 3 weeks, and 7 weeks. We cannot found high difference (even no difference) in cell viability when comparing cement and DBSs. The analysis shows increased number of viable-cells, some variations in alignment and cell morphology for all groups. This indicates continuation of tissue-fabrication and cell differentiation.

### Attachment, Cell-Activity, and Osteogenic Differentiation of iPSC-MPs

Cell attachment, differentiation, and activity/viability are very sensitive to scaffold characteristics, for example, their pore size; composition; rate-of-porosity; and shape, interconnectivity, and mechanical properties. To arrive to some high quantity of attached-cells after culturing, we have followed two protocols, controlled, and tested them as shown in Figure 4. We have noticed that the NACs is by far greater than that when using protocol 1, as illustrated in Figure 4A, and it is in harmony with yield of DBSs: See the inset in Figure 4A.

The reduced number of non-adherent cells, Figure 4B, confirms the increased cell attachment, in addition to increased fluorescence measured following incubation with PrestoBlue^®^ reagent as shown in Figure 4.

The increased cell seeding efficiency achieved with protocol 2 is similar irrespective of the scaffold group used in the optimization study, suggesting that the structural parameters of the PTSDP scaffolds investigated in this study have a negligible effect on cell attachment. Cell survival and tissue formation were estimated *via* live/dead assay. Irrespective of the scaffold group, cells display similar viability when cultured under osteogenic conditions for 3 days, 3 weeks, and 7 weeks as shown in Figure 5.

As an interesting fact, no major differences in cell viability are seen when comparing cement and DBSs, indicating that the scaffolds developed in this study are cytocompatible and can be used to culture iPSC-MPs. Figure 4B shows that poor number of non-adherent cells is found with increased cell attachment. In addition, Figure 4C illustrates that developed fluorescence-measured following incubation with PrestoBlue^®^: indicator.

We believe that PTSDP scaffolds, studied in this work, have minor impact on cell attachment. This minor effect when increased cell seeding efficiency attained with protocol 2 is similar independently of the scaffold-group used in the optimization study. Cell-survival and tissue-formation could be estimated taken into consideration live/dead assay. For all groups, cells show similar activity/viability when cultured under suitable osteogenic-conditions for a period of 3 days, 3 weeks, and 7 weeks as illustrated in Figure 5. Impressive fact is that there are important changes in cell-viability. This is seen when we compare cement- and decellularized-bone-scaffolds, indicating that the scaffolds developed in this study are cytocompatible and can be used to culture iPSC-MPs. The analysis also reveals increased cell density and changes in cell morphology and alignment for all groups investigated, indicating ongoing tissue formation and cell differentiation (Amini and Nukavarapu, 2012). We believe that PTSDP scaffolds support osteogenic differentiation of different types of stem cells. This includes human BMSCs, stem cells, deciduous teeth stem cells, transduced human-induced pluripotent, stem cell-derived mesenchymal, stromal cells (hiPSCMSCs), etc. This behavior has been observed in several published Works such as: (i) on calcium phosphate (He et al., 2013; Zhang et al., 2015; Magin et al., 2016), (ii) on human umbilical cord mesenchymal stem (MSCs) (Nagamura-Inoue and He, 2014; Lian et al., 2016) and (iii) on human embryonic stem cells (Rao et al., 2015; Kim et al., 2016).

However, no studies exist on the effects of macro PTSDP scaffolds on the osteogenic differentiation of human iPSC-MPs. Differentiation toward the osteogenic lineage is associated with the controlled up-regulation of specific genes that regulate the differentiation process and/or play a role in the formation of new tissue (Gowri et al., 2013). In addition, master transcription factor can control the expression of downstream genes in the early phase of osteogenic differentiation including COL1A1: and OPN. Because the PEG particles of 100–600 μm size create pores with wider pore size distribution, the expression of COL1A1, and OPN are highest on their scaffolds.

**FIGURE 6:**
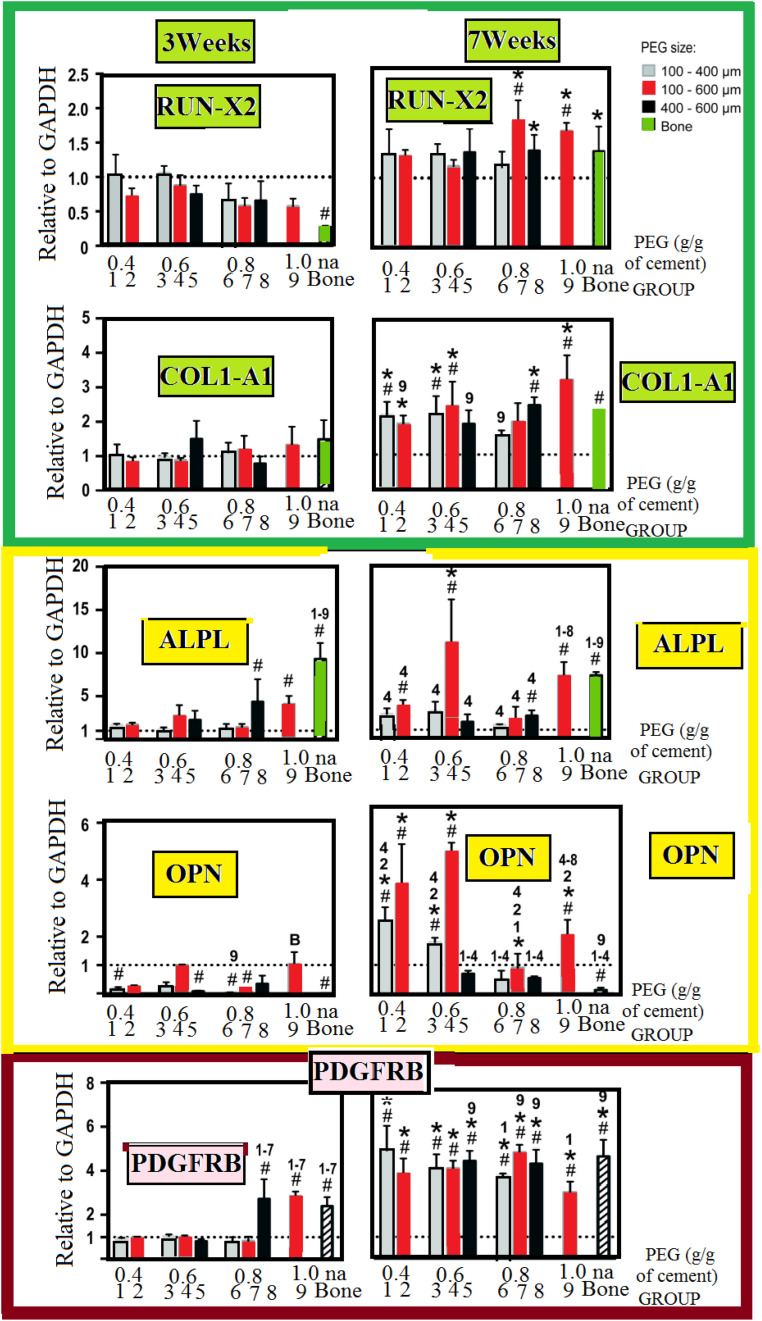
Osteogenic-contrast/gene-expression of osteogenic all along the seeding interval for 1013A-derived mesenchymal-progenitors (1013A-MPs) cultured on cement-scaffolds and DB. We present the experimental data as distributed for the expression-level. We measure these data 3 days after culturing and before osteogenic-induction which is denoted by dashed-line, and it corresponds to the expression-level of the housekeeping-gene/GAPDH. All results are shown as mean ± standard deviations (*n* = 3–4, *P* < 0.05). We use hash/tags to show an important difference to the first time (day-0), we use asterisks to stand for important change between 3 weeks and 7 weeks. Finally, we denoted by the letter B and different numbers to denote bone and different groups: Here, we notice important difference between some scaffold-groups and decellularized-bone, respectively.

We studied the osteogenic lineages using a real-time PCR, to dfferentiate between the different osteogenic lineages. This is illustrated in Figure 6. We do not find any important change in the expression-level of the analyzed genes at the third week. However, this occurred with an exception in a few cases. However, on the contradictory, we find some important change in the expression-level of the analyzed genes at the seventh week for the majority of the groups (PTSDP/scaffold groups). We noticed that for groups 5, 7, 8, and 9, the RUNX 2:-expression is greatly raised with: [*P* < 0.05] at the seventh-week compared to third week, while the expression of COL1A1: is significantly increased (*P* < 0.05) for groups 1, 2, 3, 4, 8, and 9. As interesting matter, expression of RUNX2: and COL1A1: in PTSDP scaffolds is similar to that of DB.

At the seventh weak, in G-4, the expression of ALPL and OPN is higher (*p* < 0.05) compared to the other group-scaffolds. The scaffolds with select porosity and mechanical properties could support the differentiation of 1013A-MP and they can have more mature osteoblastic cells compared to the other PTSDP groups and DBSs.

Expression of ALPL^–^ and OPN^–^, for group 4, is stronger with probability inferior than 0.05 at the seventh week. Compared to the other PTSDP groups and DBSs, suggesting that scaffolds with select porosity and mechanical properties could support the differentiation of 1013A-MP toward more mature osteoblastic cells. Expression of PDGFRB significantly increases at week 7 compared to week 3 for all PTSDP groups and DB. Finally, the expression level of PDGFRB at week 7 is comparable for all PTSDP groups (except group 9) and DBSs.

Taken together, these results show that the expression of bone-specific genes generally increases during the culture period but the level of expression is different when cells are cultured onto PTSDP scaffold groups with different porosity, and mechanical properties, indicating an effect played by the scaffold parameters on cell differentiation.

Unfortunately, it is currently unclear which particular scaffold parameter is responsible for the observed outcomes. Scaffold features can have an independent or combined effect on cell behavior, and the specific biological response to the scaffolding materials depends on the specific phenotype and stage of development of the cells tested (Kaur et al., 2010).

This complex cell-material interaction makes it difficult to draw conclusion regarding the relative contribution of each scaffold parameter on cellular response, as well as to compare studies conducted by different groups using different biomaterial scaffolds and cell lines. Systematic studies that address these issues are therefore encouraged before optimal scaffolds can be developed and used for specific bone engineering applications.

In this study, we have developed macroporous PTSDP scaffolds with an interconnected porosity that mimics the bone micro environment, with objective to engineer bone grafts with enhanced healing properties. While PEG has been used as a porogen in hydrogel and polymeric scaffolds, this study is the first report of ceramic scaffolds with PEG as a macroporous porogen tested *in vitro*. The results show that macro PTSDP scaffolds with defined structural parameters and mechanical properties can be fabricated using our engineering approach based on moldable cement paste and PEGPs as porogen.

Other significant markers with osteogenic differentiation of mesenchymal stem cells are the metalloenzyme ALPL and tyrosine kinase PDGFRB dental pulp and BMP2:RUNX2 is an early (Li et al., 2018). Differentiation toward the osteogenic lineage was studied *via* real-time PCR as shown in Figure 6. Except in a few cases, no substantial increase in the expression level of the analyzed genes is observed at week 3. In contrast, a significant increased expression is observed after week 7 for most of the PTSDP scaffold groups. For example, the expression of RUNX2: is significantly increased (*P* < 0.05) at week 7 compared to week 3 for group 5, 7, 8, and 9, while the expression of COL1A1: is significantly increased (*P* < 0.05) for groups 1, 2, 3, 4, 8, and 9. As an interesting matter, expression of RUNX2: and COL1A1: in PTSDP scaffolds is similar to that of DB. Expression of PDGFRB significantly increases at week 7 compared to week 3 for all PTSDP groups and DB. Finally, the expression level of PDGFRB at week 7 is comparable for all PTSDP groups (except group 9) and DBSs. Taken together, these results show that the expression of bone-specific genes generally increases during the culture period but the level of expression is different when cells are cultured onto PTSDP scaffold groups with different porosity, and mechanical properties, indicating an effect played by the scaffold parameters on cell differentiation. Unfortunately, it is currently unclear which particular scaffold parameter is responsible for the observed outcomes. Scaffold features can have an independent or combined effect on cell behavior, and the specific biological response to the scaffolding materials, also, depends on the specific phenotype and stage development of the cells tested (Brown and Badylak, 2014).

## Experimental Section

We have followed the same procedures and techniques as stated in our previous work stated in reference (Abdalla et al., 2018).

### Scaffold Fabrication

Porous tannin spray-dried powder (PTSDP) was mixed with hydroxyapatite (Ca_10_(PO_4_)_6_ (OH)_2_) to form a powder mixture with a composition of 80 wt.% PTSDP and 20 wt.% hydroxyapatite, prepared as previously detailed (Amaral-Labat et al., 2012). Then, the powder mixture was sieved to include only 25–75 μm diameter particles. Polyethylene glycol (PEG; 20,000 MW, Sigma, St. Louis Mo.) was melted at 100°C. Then solidified at room temperature and ground to a fine powder. The milled powder was sieved to defined size ranges of 100–400, 400–600, or 100–600 μm. The PEG powder at a certain size range was added directly to the tannin-hydroxyapatite powder at a weight ratio of 0.4, 0.6, 0.8, or 1 gram of PEG powder per gram of tannin-hydroxyapatite powder. Then, these powders (PEG & tannin hydroxyl-apatite) are mixed together in a turbula for 30 min. Crystal growth inhibitors disodium dihydrogen pyrophosphate (1% by weight) and citric acid (2.4% by weight) were added directly to the powder, along with a formalin solution (10% by weight). The macroporous scaffolds were fabricated by addition of a citric acid solution (0.5 M) to the powder at a ratio of 0.25 mL per gram of powder (irrespective of the amount of PEG). The mixture was shaken in a cap mixer for 1 min and immediately manually transferred to silicone molds (8 mm diameter × 3 mm thick) using a spatula. Within 5–7 min., the molds are transferred to Dulbecco’s phosphate buffered saline with calcium (DPBS-8662, Sigma) to allow for complete setting at 37°C for 24 h.

**FIGURE 7:**
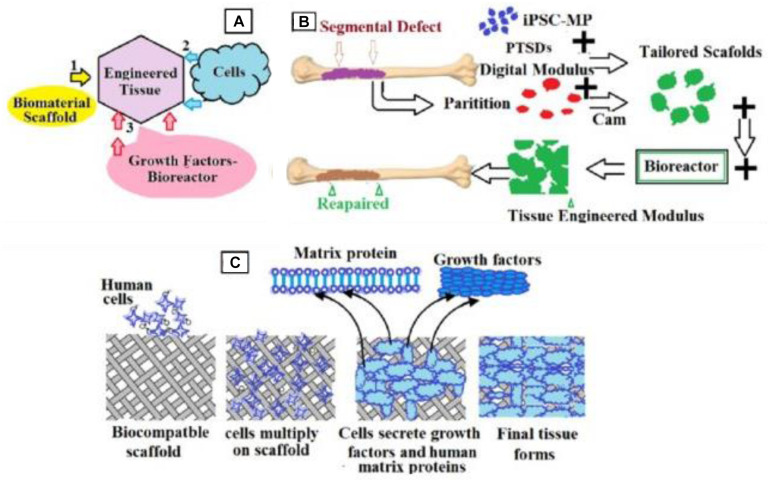
Schematic representation of the bone engineering techniques used in the present work. **(A)** Schematic representation of the engineered tissue from biomaterial scaffolds, growth factors bioreactors, and cell; **(B)** steps detailing how to obtain the manufactured tissue; **(C)** details on inserting cells into scaffolds.

Then, the samples were removed from the molds, polished with sandpaper to about 2 mm thickness, and transferred to fresh DPBS-8662 at 70°C to melt and leach the PEG for 48 h. The absence of PEG was confirmed by differential scanning calorimetry (DSC) and X-ray diffraction analysis (XRD) as described below. Each sample was dried at 70°C, for 16 h, then sterilized in an autoclave at 150°C for 30 min and used for characterization and cell culture (Figure 7). Ten scaffold groups were thus manufactured. Bone disks were prepared as previously described (de Peppo et al., 2013) incorporated herein by reference in its entirety]. Plugs of trabecular bone (8 mm diameter) were drilled from the sub-chondral region of meta-carpal joints of calves (Green Village Packing, Green Village, NJ, United States). Soon after, plugs were cleansed under a high-pressure water stream to remove the bone marrow. Then, we sequentially washed the plugs with a solution of ethylenediamine tetra-acetic acid (EDTA) (0.1%) in DPBS-8662, then EDTA (0.1%) in Tris (10 mM), then SDS (0.5%) in Tris (10 mM), followed by treatment with a solution of DNase and RNase in Tris–buffer (10 mM) to remove cellular material. DB plugs were thoroughly rinsed in DPBS-8662, freeze-dried, cut, and then polished to about 2 mm thickness. Each individual scaffold was weighed and measured to calculate the density, and those in the range of 0.27–0.35 g/cm^3^ were used for material characterization analysis and cell culture. For cell culture, scaffolds were selected and sterilized overnight in ethanol (70% by volume); and then conditioned in expansion medium overnight.

### XRD Data

**FIGURE 8:**
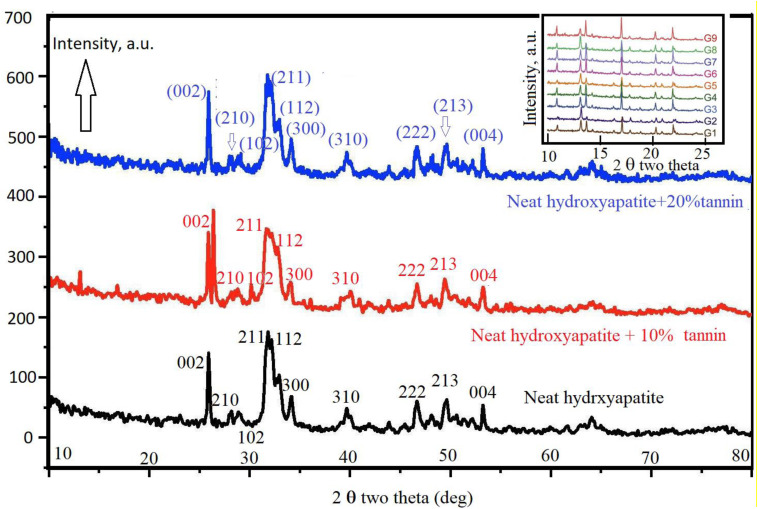
Shows the XRD data of neat hydroxyapatite (black curve). Red curve shows the XRD data of hydroxyapatite with 10% tannin and blue curve represent the XRD data of hydroxyapatite with 10% tannin.

Figure 8 shows the XRD data of neat hydroxyapatite (black curve), hydroxyapatite with 10% tannin (red curve), and hydroxyapatite with 20% tannin (blue curve). The inset in this figure illustrates the XRD data of all nine groups (G1–G9). In general, the peaks shown in all XRD data indicate no presence of impurities. We use Debye–Scherrer’s equation to estimate the average crystallite size of the three samples. We note broad peak in the range of 30 < 2θ < 40 which are attributed to (211), (112), (300), and (202) reflections of hydroxyapatite. The X-ray data confirm that the crystallinity increases with the addition of tannin in accordance with the FTIR data. In addition, the XRD data show that addition of tannin changes the crystal planes. The inset in Figure 8 shows the XRD data for the studied nine groups.

### TEM Data

**FIGURE 9:**
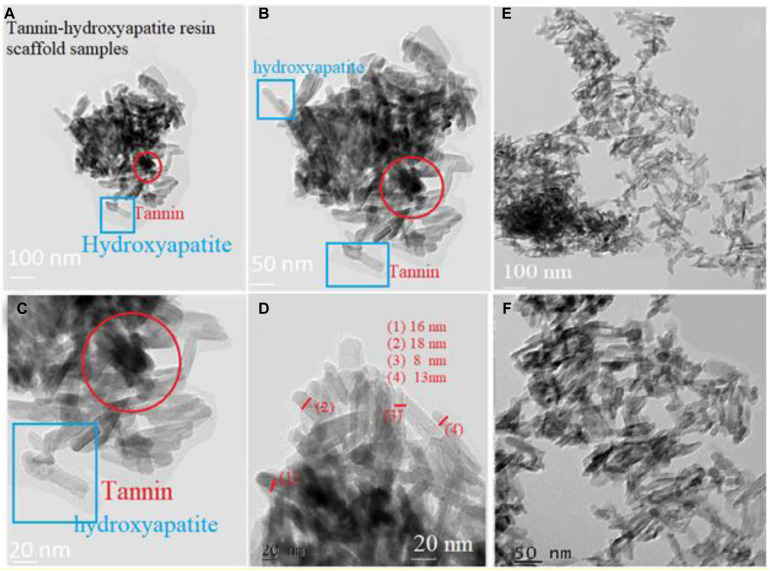
Panels **(A–D)** show the nano-rods of hydroxyapatite stick with 20% tannin. They are have irregular size and shape. In addition to some agglomeration near the marked red circle. Panels **(E,F)** illustrate hydroxyapatite stick with 10% tannin.

Figures 9a–d show the nano-rods of hydroxyapatite stick with 20% tannin. They have irregular size and shape, in addition to some agglomeration near the marked red circle. While Figures 9e,f illustrate hydroxyapatite stick with 10% tannin with good rods shape, these rods are shown imperfect and unclear in Figures 9a–d. This occurs for each PEGP size, because the morphology decrease with increasing the size of PEGPs for any PEG content. This is correct for all groups except for the scaffold groups manufactured using 0.8 g of PEG/g of cement (groups 6–8 Figures 1G–I). The size obtained from Figure 9c agrees well with XRD-data (Figure 8). The crystallite size of hydroxyapatite with 20% tannin is about 29 nm, while the crystallite size of hydroxyapatite with 10% tannin is about 24 nm, with 10% tannin (Figures 9e,f). The TEM data agree with the XRD data Fgure 8. One can notice some agglomeration in Figures 9a–d rather than in Figures 9e,f. Therefore, one can conclude that addition of tannin increases the ability of agglomeration of hydroxyapatite rods; this occurs perhaps due to the creation of extra electric charges coming from tannin and injected into the hydroxyapatite matrix. In this case, one considers tannin as a modifier to control the morphology and the shape of hydroxyapatite rods. Wang et al. (2007) have confirmed that some of the organic modifiers such as ethylene di amine tetra acetic acid ETDA can control the morphology and shape of hydroxyapatite rods.

### FTIR Data

**FIGURE 10:**
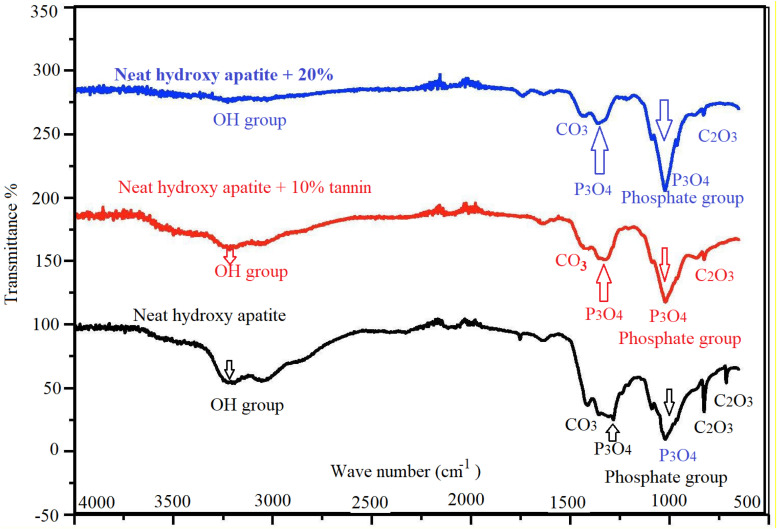
FTIR spectra of hydroxyapatite with tannin nanoparticles. Neat hydroxyapatite (black), hydroxyapatite with 10% tannin (red), and hydroxyapatite with 20% tannin (blue).

We have used FTIR technique to identify the function groups in the different scaffolds. Figure 10 illustrates that tannin plays an important role in the amelioration of the morphology of hydroxyapatite. At 1286 cm^–1^ and 1025 cm^–1^, there are two small peaks corresponding to phosphate group (P_3_O_4_). Two peaks at 3225 and 3058 cm^–1^, confirm the presence of a hydroxyl group (due to humidity content). A peak at 1411 cm^–1^ indicates the presence of carbonate group C_2_O_3_. At 2097 cm^–1^ there is a faint peak that deals with asymmetric stretching of carbonate group.

### Cell Morphology and Alignment for All Groups

In any case, whether the extracellular vesicles are present or not, two dimension culture system revealed an importantly better condition for cell proliferation than three dimension condition after 3, 21 and 49 days (*P* < 0.01). An important increase in viable cells in various conditions is recorded compared to the matched groups after the third or seventh days (*P* < 0.01). This explains that the scaffolds are not toxic and protected the cell proliferation.

## Data Availability Statement

The datasets generated during and/or analyzed during the current study are available from the corresponding author.

## Author Contributions

YY performed project planning, conducted experiments, data collection, analysis and interpretation, and drafted the manuscript. SA performed project planning, conducted experiments, data collection, analysis and interpretation, drafted the manuscript, prepared the materials, participated in all characterization, scanning, analysis, and revised the manuscript. Both authors contributed to the data analysis, interpretation, revision of the manuscript and read and approved the final manuscript.

## Conflict of Interest

The authors declare that the research was conducted in the absence of any commercial or financial relationships that could be construed as a potential conflict of interest.

## References

[B1] Abdalla S. Al-Marzouki F. Pizzi A. Bahabri F. (2018). *Bone Graft with a Tannin-hydroxyapatite Scaffold and Stem Cells for Bone Engineering*, U.S. Patent number: 10155069. Washington, DC: U.S. Patent and Trademark Office.

[B2] Abdalla S. Pizzi A. Bahabri F. Ganash A. (2015). MALDI-TOF and C13 NMR analysis of Valonea oak (*Quercus aegylops*) aicorn tannin and its adhesive application. *Bioresources* 10 5233–5244.

[B3] Al-Marzouki F. Zahed A. Pizzi A. Abdalla S. (2017). *Thermo-set Resin Composition for Brake Pads, Method of Preparation, and Brake Pad Assembly*, Patent number: 9791012. Washington, DC: U.S. Patent and Trademark Office.

[B4] Al-Munajjed A. A. Hien M. Kujat R. Gleeson J. P. Hammer J. (2008). Influence of pore size on tensile strength, permeability and porosity of hyaluronan-collagen scaffolds. *J. Mater. Sci. Mater. Med.* 19 2859–2864. 10.1007/s10856-008-3422-5 18347950

[B5] Amaral-Labat G. Grishechko L. Szczurek A. Fierro V. Pizzi A. Kuznetsov B. (2012). Highly mesoporous organic aerogels derived from soy and tannin. *Green Chem.* 14 3099–3106.

[B6] Amaral-Labat G. Grishechko L. I. Fierro V. Kuznetsov B. N. Pizzi A. Celzard A. (2013). Tannin-based xerogels with distinctive porous structures. *Biomass Bio Energy* 56 437–445. 10.1016/j.biombioe.2013.06

[B7] Amini A. R. Nukavarapu S. P. (2012). Bone tissue engineering: recent advances and challenges. *Crit. Rev. Biomed. Eng.* 40 363–408. 10.1615/critrevbiomedeng.v40.i5.10 23339648 PMC3766369

[B8] Basso M. C. Giovando S. Pizzi A. (2014a). *Composition for Manufacturing an Elastic Tannin based Foam Material, and process Thereof*, Publication Number WO2014117946 A1, Publication date Aug 7, 2014, Application number PCT/EP2014/000271, 2014. Available online at: http://patents.justia.com/inventor/antonio-pizzi (accessed December 17, 2015).

[B9] Basso M. C. Lagel M. C. Pizzi A. Celzard A. Abdalla S. (2015). First tools for tannin-furanic foams design. *Bioresources* 10 5233–5241. 10.15376/biores.10.3.5233-5241

[B10] Basso M. C. Pizzi A. Lacoste C. Delmotte L. Al-Marzouki F. Abdalla S. (2014b). Tannin-furanic-polyurethane foams for industrial continuous plant lines. *Polymers* 6 2985–3004.

[B11] Brown N. B. Badylak S. F. (2014). Extracellular matrix as an inductive scaffold for functional tissue reconstruction. *Transl. Res.* 163 268–285. 10.1016/j.trsl.2013.11.003 24291155 PMC4203714

[B12] Celzard A. Basso M. C. Pizzi A. Fierro V. (2013). *Rigid Foams based On Procyanidin- And/Or Prodelphinidin-Type Tannins And Preparation Method There of* Publication number: 20140193322. Available online at: https://patentscope.wipo.int/search/en/detail.jsf?docId=WO2013026974 (accessed February 28, 2013).

[B13] Celzard A. Szczurek A. Fierro V. Pizzi A. (2015). *Cellular Porous Monoliths Containing Condensed Tannins*, Publication number: 20150274921. Available online at: http://patents.justia.com/inventor/antonio-pizzi (accessed June 12, 2018).

[B14] Chen Q. Baino F. Spriano S. Pugno N. M. Vitale-Brovarone C. (2014). Modelling of the strength–porosity relationship in glass-ceramic foam scaffolds for bone repair. *J. Eur. Ceramic Soc.* 34 2663–2673. 10.1016/j.jeurceramsoc.2013.11.041

[B15] Chen T. H. Ghayor C. Siegenthaler B. Schuler F. Rüegg J. De Wild M. (2018). Lattice micro architecture for bone tissue engineering from calcium phosphate compared to titanium. *Tissue Eng. Part A* 24 1554–1561. 10.1089/ten.tea.2018.0014 29999466 PMC6198759

[B16] Clegg A. Young J. Iliffe S. Rockwood K. (2013). Frailty in elderly people. *Lancet* 381 752–762. 23395245 10.1016/S0140-6736(12)62167-9 PMC4098658

[B17] Crupi V. Jannelli M. P. Magazu S. Maisano G. Majolino D. Migliardo P. (1999). Raman spectroscopic study of water in the poly(ethylene glycol) hydration shell. *J. Mol. Struct.* 381 207–212. 10.1016/0022-2860(96)09308-8

[B18] de Peppo G. M. Marcos-Campos I. Kahler D. J. Alsalman D. Shang L. Vunjak-Novakovic G. (2013). Engineering bone tissue substitutes from human induced pluripotent stem cells. *Proc. Natl. Acad. Sci. U.S.A.* 110 8680–8685. 23653480 10.1073/pnas.1301190110 PMC3666730

[B19] Gowri A. M. Kavitha G. Rajasundari M. Mubeen F. Raj G. D. (2013). Foetal stem cell derivation & characterization for osteogenic lineage. *Indian J. Med. Res.* 137 308–315. 23563374 PMC3657854

[B20] Guo J. Sun W. Kim J. P. Lu X. Li Q. Lin M. (2018). Development of tannin-inspired antimicrobial bio adhesives. *Acta Biomaterialia* 72 35–44. 29555464 10.1016/j.actbio.2018.03.008 PMC6328059

[B21] He F. Li J. Ye J. (2013). Improvement of cell response of the poly(lactic-co-glycolic acid)/calcium phosphate cement composite scaffold with unidirectional pore structure by the surface immobilization of collagen via plasma treatment. *Colloids Surf. B Biointerfaces* 2013 209–216. 10.1016/j.colsurfb.2012.10.018 23201739

[B22] Hing K. A. (2004). Bone repair in the twenty-first century: biology, chemistry or engineering? *Philos. Transact. A Math. Phys. Eng. Sci.* 362:2821. 10.1098/rsta.2004.1466 15539372

[B23] Jeonga C. G. Hollister S. J. (2010). Mechanical, permeability, and degradation properties of 3D designed poly (1, 8 Octanediol-co-Citrate) (POC) scaffolds for soft tissue engineering. *J. Biomed. Mater. Res. B Appl. Biomater.* 93 141–149. 10.1002/jbm.b.31568 20091910 PMC4369673

[B24] Kaur G. Valarmathi M. T. Potts J. D. Jabbari E. Sabo-Attwood T. Wang Q. (2010). Regulation of osteogenic differentiation of rat bone marrow stromal cells on 2D nano rod substrates. *Biomaterials* 31 1732–1741. 10.1016/j.biomaterials.2009.11.041 20022632 PMC4748726

[B25] Khaledi A. A. R. Vojdani M. Farzin M. Pirouzi S. (2018). The effect of sintering program on the compressive strength of zirconia copings. *J. Dent. (Shiraz)* 19 206–211. 30175190 PMC6092466

[B26] Khaledi A. A. R. Vojdani M. Farzin M. Pirouzi S. Orandi S. (2019). The effect of sintering time on the marginal fit of zirconia copings. *J. Prosthodont.* 28 e285–e289. 10.1111/jopr.12731 29314433

[B27] Kim H. D. Lee E. A. Choi Y. H. An Y. H. Koh R. H. Kim S. L. (2016). High throughput approaches for controlled stem cell differentiation. *Acta Biomater.* 1 21–29. 10.1016/j.actbio.2016.02.022 26884279

[B28] Kim J. J. (2015). Applications of iPSCs in cancer research. *Biomark Insights* 10(Suppl. 1) 125–131. 10.4137/BMI.S20065 PMC4521640 26279620

[B29] Konai N. Pizzi A. Raidandi D. Lagel M. C. Saidou C. Hamido A. (2015). Aningre tannin extract characterization and performance as an adhesive resin. *Ind. Crops Prods.* 77 225–231. 10.1016/j.indcrop.2015.08.053

[B30] Lacoste C. Basso M. C. Pizzi A. Laborie M. P. Celzard A. Fierro V. (2013). Chemical modification of tannins to elaborate aromatic bio based macromolecular architectures. *Ind. Crops Prods.* 43 245–250.

[B31] Lagel M. C. Pizzi A. Basso M. C. Delmotte L. Abdalla S. Zahed A. (2016). Automotive brake pads made with a bio resin matrix. *Ind. Crops Prods.* 85 372–381. 10.1016/j.indcrop.2015.12.090

[B32] Lee J. B. Maeng W. Y. Koh Y. H. Kim H. E. (2018). Porous calcium phosphate ceramic scaffolds with tailored pore orientations and mechanical properties using lithography-based ceramic 3D printing technique. *Materials (Basel)* 11:1711. 10.3390/ma11091711 30217045 PMC6164124

[B33] Li B. Gao Y. Guo L. Fan Y. Kawazoe N. Fan H. (2018). Synthesis of photo-reactive poly (vinyl alcohol) and construction of scaffold-free cartilage like pellets in vitro. *Regen. Biomater.* 5 159–166. 10.1093/rb/rby009 29942648 PMC6007571

[B34] Li X. Pizzi A. Cangemi M. Fierro V. Celzard A. (2012). Flexible natural tannin-based and protein-based bio-sourced foams. *Ind. Crops Prods.* 37 389–393. 10.1016/j.indcrop.2011.12.037

[B35] Lian J. Lv S. Liu C. Liu Y. Wang S. Guo X. (2016). Effects of serial passage on the characteristics and cardiac and neural differentiation of human umbilical cord Wharton’s jelly-derived mesenchymal stem cells. *Stem Cells Int.* 2016:9291013. 10.1155/2016/9291013 26798365 PMC4699056

[B36] Lin Y. Umebayashi M. Abdallah M.N. Dong G. Roskies M.G. Zhao Y.F. (2019). Combination of polyether-ketoneketone scaffold and human mesenchymal stem cells from temporo-mandibular joint synovial fluid enhances bone regeneration. *Sci. Rep.* 9:472 10.1038/s41598-018-36778-2 PMC6345789 30679553

[B37] Magin C. M. Alge D. L. Anseth K. S. (2016). Bio-inspired 3D microenvironments: a new dimension in tissue engineering. *Biomed. Mater.* 11:022001 10.1088/1748-6041/11/2/022001 PMC11938994 26942469

[B38] Nagamura-Inoue T. He H. (2014). Umbilical cord-derived mesenchymal stem cells: their advantages and potential clinical utility. *World J. Stem Cells* 6 195–202. 24772246 10.4252/wjsc.v6.i2.195 PMC3999777

[B39] Natarajan V. Krithica N. Madhan B. Sehgal P. K. (2012). Preparation and properties of tannic acid cross-linked collagen scaffold and its application in wound healing. *J. Biomed. Mater. Res. B Appl. Biomater.* 101 560–567. 10.1002/jbm.b.32856 23255343

[B40] Oreffo R. O. Triffitt J. T. (1999). Future potentials for using osteogenic stem cells and biomaterials in orthopedics. *Bone* 25(2 Suppl) 5S–9S. 10.1016/s8756-3282(99)00124-6 10458266

[B41] Park S. Whittington C. Voytik-Harbin S. L. Han B. (2015). Microstructural parameter-based modeling for transport properties of collagen matrices. *J. Biomech. Eng.* 137:061003. 10.1115/1.4029920 PMC4403517 25728145

[B42] Pennella F. Cerino G. Massai D. Gallo D. Falvo D. Urso Labate G. (2013). A survey of methods for the evaluation of tissue engineering scaffold permeability. *Ann. Biomed. Eng.* 41 2027–2041. 10.1007/s10439-013-0815-5 23612914

[B43] Pizzi A. Basso M. C. Celzard A. Fierro V. Giova S. (2013). *Process of Recycling Leather Residues and Production of Composite Materials*, Publication number WO2013010668 A1, Application number, PCT/EP2012/003031.

[B44] Rao V. Shih Yu-Ru V. Kang H. Kabra H. Varghese S. (2015). Adenosine signaling mediates osteogenic differentiation of human embryonic stem cells on mineralized matrices. *Front. Bioeng. Biotechnol.* 2015:185. 10.3389/fbioe.2015.00185 26618155 PMC4639610

[B45] Stern R. Kogan G. Jedrzejas M. J. Soltés L. (2007). The many ways to cleave hyaluronan. *Biotechnol. Adv.* 25 537–557. 10.1016/j.biotechadv.2007.07.001 17716848

[B46] Takahashi K. Tanabe K. Ohnuki M. Narita M. Ichisaka T. Tomoda K. (2007). Induction of pluripotent stem cells from adult human fibroblasts by defined factors. *Cell* 131 861–872. 10.1016/j.cell.2007.11.019 18035408

[B47] Tondi G. Pizzi A. (2009). Tannin-based rigid foams: characterization and modification. *Ind. Crops Prods.* 29 356–363. 10.1016/j.indcrop.2008.07.003

[B48] U.S. Food and Drug Administration (2019). *CFR – Code of Federal Regulations Title 21.* Available online at: https://www.accessdata.fda.gov/scripts/cdrh/cfdocs/cfcfr/CFRSearch.cfm?CFRPart=184&showFR=1

[B49] Vu L. T. Jain G. Veres B. D. Rajagopalan P. (2015). Cell migration on planar and three-dimensional matrices: a hydrogel-based perspective. *Tissue Eng. Part B Rev.* 21 67–74. 25011932 10.1089/ten.teb.2013.0782 PMC4321976

[B50] Wang A. Liu D. Yin H. Wu H. Wada Y. Ren M. (2007). Size-controlled synthesis of hydroxyapatite nanorods by chemical precipitation in the presence of organic modifiers. *Mater. Sci. Eng. C* 27 865–869. 10.1016/j.msec.2006.10.001

[B51] Xu Q. Ensign L. M. Boylan N. J. Hanes J. (2015). Impact of surface polyethylene glycol (PEG) density on biodegradable nanoparticle transport in mucus ex vivo and distribution in vivo. *ACS Nano* 9 9217–9227. 10.1021/acsnano.5b03876 26301576 PMC4890729

[B52] Yu J. Hu K. Smuga-Otto K. Tian S. Stewart R. Slukvin I. I. (2009). Human induced pluripotent stem cells free of vector and trans-gene sequences. *Science* 324 797–801. 10.1126/science.1172482 19325077 PMC2758053

[B53] Zhang H. X. Xiao G. Y. Wang X. Dong Z. G. Ma Z. Y. Li L. (2015). Biocompatibility and osteogenesis of calcium phosphate composite scaffolds containing simvastatin-loaded PLGA microspheres for bone tissue engineering. *J. Biomed. Mater. Res. A* 103 3250–3258. 10.1002/jbm.a.35463 25809455

[B54] Zhang K. Fan Y. Dunne N. Li X. (2018). Effect of micro-porosity on scaffolds for bone tissue engineering. *Regen. Biomater.* 5 115–124. 10.1093/rb/rby001 29644093 PMC5887944

